# Graphene Oxide Nanofluids for Heavy-Oil Recovery: Experimental Evaluation and Field-Scale Numerical Simulation of Recovery Potential

**DOI:** 10.1021/acsomega.5c10260

**Published:** 2026-01-22

**Authors:** Jimena Gómez-Delgado, Andres Felipe Ortiz, Javier Jaimes, Raúl Andrés Martinez-Lopez, Nicolás Santos-Santos, Enrique Mejía-Ospino

**Affiliations:** † Grupo de Investigación en Tomografía Cumputarizada para Caracterización de Yacimientos (GIT), 28014 Universidad Industrial de Santander, 680002 Bucaramanga, Colombia; ‡ Laboratorio de Espectroscopia Atómica y Molecular (LEAM), 28014 Universidad Industrial de Santander, 680002 Bucaramanga, Colombia

## Abstract

Increasing the recovery factor in oil fields is a critical task for improving reservoir performance and energy sustainability. This study investigates the novel application of graphene oxide (GO) nanoparticles as an enhanced oil recovery (EOR) agent in heavy oilfields, with an integrated multiscale approach combining laboratory experiments and numerical reservoir simulation. The nanofluids were optimized by evaluating the influence of salinity (300–900 ppm), pH (4–8), and GO concentrations (0.03–0.09 wt %) on interfacial tension (IFT) and wettability. Under optimal conditions (900 ppm brine, pH 8, and 0.09 wt % GO), the IFT decreased from 32.5 to 15.8 mN/m, and the contact angle shifted from 140° (oil-wet) to 90° (intermediate). Coreflooding tests confirmed the EOR potential of GO nanofluids, achieving 63.60% oil recovery compared to 56.72% with conventional waterflooding, an incremental gain of 7%. Relative permeability curves and advanced wettability indices (Lak and modified Lak) validated wettability alteration effects. To evaluate the scalability of this technology, the experimental data were incorporated into a numerical simulation using CMG-STARS. First, a history-matched core-scale model was developed to reproduce laboratory results. Then, a conceptual reservoir model was constructed using representative petrophysical properties from Colombian fields. The reservoir-scale simulation showed that nano-GO injection could yield an additional 402,431 barrels of oil over a 20-year period compared to conventional waterflooding, while maintaining a more favorable water cut. These findings highlight the potential of GO nanofluids as a viable and scalable EOR strategy for heavy-oil reservoirs. Future studies will focus on field-scale validation, economic feasibility, and environmental impact.

## Graphical abstract

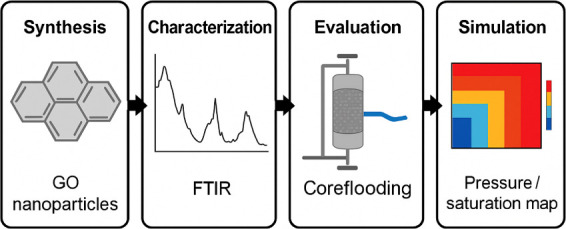

## 1 Introduction

In contrast to the widespread global adoption of EOR techniques, Colombia faces a significant challenge with its oil recovery factor at a modest 19%, substantially below the global average of 37%.^1^ This disparity underscores the critical need for innovative EOR solutions in the Colombian oil industry. EOR techniques aim to increase the amount of crude oil extracted from an oil field beyond what is achievable through primary and secondary recovery methods. These techniques typically involve introducing materials not normally present in the reservoir to increase oil mobility and extraction efficiency.

The potential for improvement in Colombia is substantial, considering that approximately 70% of the country’s oil fields have been producing for over 30 years.^2^ As of 2010, only 23 commercial applications of secondary recovery techniques existed in Colombia, with even fewer tertiary recovery applications.^3^ This limited implementation of advanced recovery methods presents a significant opportunity for enhancing the country’s petroleum resource extraction efficiency.

Nanotechnology has emerged as a promising frontier in EOR, offering potential solutions to the challenges faced by conventional methods. Nanoparticles, with their unique properties at the nanoscale, have garnered considerable attention for their ability to optimize oil recovery processes.^4^ Among these, GO nanoparticles stand out due to their exceptional characteristics, including high surface area, electrical conductivity, and mechanical strength. These intrinsic properties are relevant to EOR because the high specific surface area provides abundant active sites for adsorption at the oil–water and rock–fluid interfaces, supporting mechanisms such as wettability alteration and interfacial tension reduction. Additionally, the mechanical strength and structural stability of the GO nanosheets help maintain dispersion integrity during flow through porous media, preventing aggregation and enabling effective nanoparticle transport.^5^

The use of nanoparticles in EOR addresses several limitations of traditional recovery agents. Unlike polymer flooding, which can face issues with mechanical degradation and high costs, nanoparticles offer improved stability and cost-effectiveness. In this context, mechanical degradation refers to shear-induced breakdown of polymer chains during injection, which decreases viscosity and reduces sweep efficiency. Moreover, polymer flooding often suffers from additional limitations such as poor injectivity, thermal and chemical instability, sensitivity to salinity, and adsorption onto mineral surfaces, all of which can compromise field performance.^6^ Surfactants, while effective in reducing interfacial tension, often suffer from adsorption onto rock surfaces, reducing their efficiency. Nanoparticles, particularly GO, have demonstrated superior performance in altering wettability and reducing interfacial tension, even at low concentrations.^7^

Previous studies have explored various nanoparticles for EOR, including silica oxide, zirconium dioxide, and aluminum oxide.^8^ However, GO nanoparticles offer unique advantages. Their two-dimensional structure and amphiphilic nature allow for more effective interaction with both the oil and water phases, potentially leading to improved oil displacement.^9^ Additionally, GO nanoparticles have shown superior wettability alteration efficiency compared to common nanoparticles, particularly in ultralow-permeability reservoirs.^10^

Recent studies have further demonstrated the potential of graphene-based nanomaterials in EOR applications. Liu et al.^11^ investigated an amphiphilic graphene oxide (GOA) derivative, which showed excellent dispersibility and stability in brine, and effectively decreased interfacial energy at concentrations as low as 45 mg/L. The study reported an additional oil recovery factor of 18.2% of the original oil in place at a GOA concentration of 100 mg/L, highlighting its cost-effectiveness in chemical EOR.

Aliabadian et al.^12^ explored the use of graphene oxide nanofillers in combination with partially hydrolyzed polyacrylamide (HPAM) solutions for heavy-oil recovery. Their findings showed that incorporating 0.2 wt % of graphene oxide with OH group functionalization mainly on the basal plane improved oil recovery by 7.8% compared to HPAM alone. This study underscores the potential synergistic effects of combining graphene oxide with traditional EOR agents.

Furthermore, Cao et al.^13^ developed a polyoxyethylated graphene oxide-based nanofluid (P-GO-O) that exhibited excellent high temperature and high salinity resistance. The P-GO-O nanofluid demonstrated the ability to lower oil–water interfacial tension to 12.2 mN/m and alter wettability from oil-wet to water-wet. In oil displacement tests, the P-GO-O nanofluid achieved a 17.2% oil recovery ratio, outperforming other graphene oxide-based nanofluids.

These studies collectively highlight the diverse mechanisms through which graphene oxide-based nanomaterials can enhance oil recovery, including interfacial tension reduction, wettability alteration, and improved stability under reservoir conditions.^14,15^

Despite these promising characteristics, the application of GO nanoparticles in EOR, especially in the context of Colombian heavy-oil fields, remains understudied. The influence of factors such as the concentration, pH, and salinity on GO nanofluid performance in heavy-oil recovery has not been systematically investigated. This research gap presents an opportunity to optimize GO nanofluid formulations for the specific conditions of Colombian oil reservoirs.

This study addresses these knowledge gaps through a comprehensive, multiscale evaluation of GO nanofluids for EOR. At the laboratory scale, the research investigates the effects of GO concentration, salinity, and pH on interfacial tension, wettability, and displacement efficiency, including coreflooding tests under reservoir-representative conditions. Furthermore, the experimental findings are integrated into a numerical simulation framework using CMG-STARS, allowing for history-matched modeling of laboratory results and extrapolation to a conceptual reservoir model. This dual approach enables prediction of oil recovery gains at the field scale, providing actionable insights for the design of pilot implementations.

The novelty of this work lies in its integration of experimental and numerical methods to evaluate the technical feasibility of GO-based EOR solutions. By bridging the gap between laboratory performance and reservoir-scale application, this study contributes to both the fundamental understanding of GO nanoparticle behavior in porous media and the development of scalable, efficient EOR strategies for challenging heavy-oil reservoirs in Colombia and beyond.

## 2 Methodologies

### 2.1 Materials

Graphite powder, sodium nitrate, potassium permanganate (99%), sulfuric acid (98%), hydrochloric acid, hydrogen peroxide solution, pure sodium chloride, sodium hydroxide, and toluene were obtained from Merck. The oil samples and sandstone were collected from an oil field located in the northeastern Colombia. The API gravity of the crude oil, SARA (saturates, aromatics, resins, and asphaltenes) analysis, and the formation brine composition are detailed in Table 1.

**Table 1:** Field Fluid Properties

formation brine					
Na^+^ (mg/L)	K^+^ (mg/L)	Mg^2+^ (mg/L)	Ca^2+^ (mg/L)	Sr^2+^ (mg/L)	Ba^2+^ (mg/L)
2440.65	17.40	40.20	345.80	7.80	15.70
crude oil					
API gravity (°)	density (g/mL)	saturated (wt %)	aromatic (wt %)	resins (wt %)	asphaltenes (wt %)
19.5	1.067	30.17	25.8	22.4	12.3

### 2.2 Sandstone Sample

A sandstone core was used, and petrophysical properties of the formation plug were determined by a fully automated Vinci Technologies multisample porosimeter-permeameter, which was used with helium and nitrogen at different confining pressures ranging from 400 to 10,000 psi. A core plug was cut by a core plugging machine, and the sample was cleaned by the Dean–Stark method using toluene; then, the sample was dried at 120 °C in an oven for 24 h. The petrophysical properties of the sample are listed in Table 2. An XRD analysis was performed to have the composition of the rock; in Figure 1, the presence of quartz can be confirmed in a majority of 75% with some traces of clay minerals.

**Table 2:**
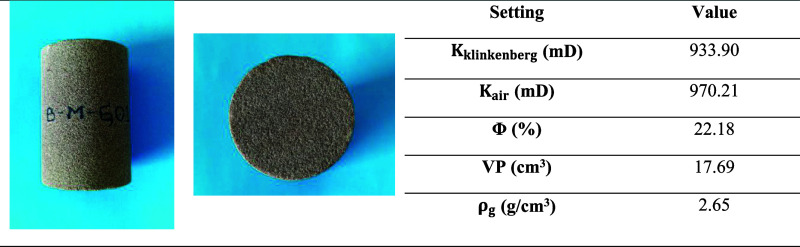
Petrophysical Properties^a^

a Photographs taken by the authors.

**Figure 1:**
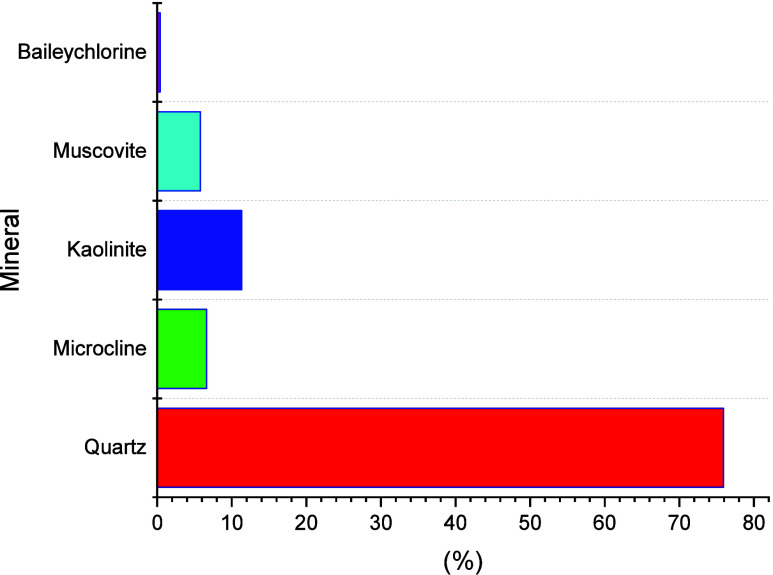
X-ray diffraction analysis of the core plug sample.

### 2.3 Fabrication of the GO Nanofluid

The nanosheets composed of GO were synthesized using a modified version of the Tour’s method based on the Hummers technique.^16^ In summary, a mixture was prepared by combining 2 g of pure graphite powder with 120 mL of sulfuric acid in a container, where it underwent constant stirring at 60 °C. Following this, potassium permanganate was introduced gradually at a rate of 0.5 g per minute, all while ensuring the temperature remained constant at 60 °C. This process was sustained for 24 h. Afterward, a small amount of 30% hydrogen peroxide was carefully added drop by drop to halt the oxidation process. Next, 500 mL of purified water was mixed in, and the solution was left undisturbed at room temperature for 8 h. Through repeated washing and centrifugation at 8000 rpm for 30 min, any remaining acids and oxidizing agents were eliminated from the GO nanosheets, adjusting the pH to 8 by adding 0.1 M NaOH. Finally, the resulting GO was combined with 500 mL of purified water and subjected to ultrasonic exfoliation for 6 h, producing a GO aqueous dispersion.^17^ To ensure that the dispersion did not clog the porous medium or damage the formation, it was filtered through a 45 μm cellulose membrane. Finally, the GO dispersion was freeze-dried at −30 °C for 5 days, followed by lyophilization to obtain the GO powder.

### 2.4 Characterization of the Materials

Fourier transform infrared spectra (FTIR) of the GO were recorded in the scanning range of 4000–500 cm^–1^. FTIR spectra were obtained using a Nicolet iS50 Thermo Scientific spectrophotometer. The morphology and structure of GO were investigated by transmission electron microscopy (TEM) using a JEM-1210 (JEOL) microscope operated at 120 kV. Thermogravimetric analysis (TGA) was used to analyze the thermal decomposition processes of GO using an STA 449 F3 Jupiter (NETZSCH) equipment, operating in the range of 32–1000 °C at a rate of 15 °C/min. The size distribution and stability of the prepared GO nanofluid were investigated using dynamic light scattering (DLS) measurement and zeta potential, respectively. The sandstone core samples were subjected to X-ray diffraction (XRD) and scanning electron microscopy (SEM) to provide micrographs illustrating the morphology and nature of the samples before and after coreflooding testing.

### 2.5 Preparation of Nanofluids

Three different concentrations of GO were chosen to prepare nanofluids (0.03, 0.06, and 0.09 wt %), which were adjusted to pH 4, 6, and 8 due to the fact that the stability of GO at different pH levels is influenced by its surface charge and interactions with surrounding ions.^18–21^ At high pH, the carboxylic acid groups on the surface of GO become ionized (deprotonated), leading to a negatively charged surface. This negative charge on the GO surface creates electrostatic repulsion between adjacent GO sheets, preventing their aggregation and promoting stability in the solution.^10^

Initially, a stability curve was constructed at various concentrations of formation brine, revealing that GO alone exhibited stability at brine concentrations below 1000 ppm. Considering this, a concentration of 900 ppm was selected for the formulation of the nanofluids. This choice stemmed from the observation that, at concentrations exceeding 1000 ppm, GO tended to form micelles and precipitate. Subsequently, the nanofluids were prepared by homogenizing the selected 900 ppm formulation of formation brine under ultrasonic conditions for 60 min. The viscosity of the GO nanofluid (900 ppm GO in 900 ppm brine at pH 8) was measured at 52 °C, showing no significant deviation from the viscosity of the base brine.

### 2.6 Measurement of Interfacial Tension (IFT) and Contact Angle

IFT measurements were conducted using the pendant drop method with a 752 × 582-pixel camera in a KRÜSS equipment. In this approach, a needle is utilized to introduce a droplet of crude oil at its tip. A container filled with the nanofluid is placed beneath a powerful light source, positioned separately from the camera. After injecting the crude oil droplet into the nanofluid, the camera records the droplet’s shape. The captured image is then analyzed to measure the IFT. The pendant drop under steady-state conditions follows the Young–Laplace equation (eq 1).

$$\gamma \left(\frac{1}{R_{1}}+\frac{1}{R_{2}}\right)=\Delta P≡\Delta P_{0}-\Delta \rho qD$$ 1

In this context, *R*_1_ and *R*_2_ denote the principal ratio of curvature, γ represents the surface tension, and Δ*P* stands for the pressure difference across the drop interface. When taking the drop apex as the point of reference, Δ*P*_0_ signifies the pressure difference at this reference point, and Δρ*qD* represents the hydrostatic pressure variation.

Contact angle measurements were performed using a DSA25E KRÜSS equipment employing the captive drop method. Prior to these measurements, a wettability restoration process was conducted on the rock samples. This process involved the following steps: The sandstone rock samples were first cleaned using acetone and methanol and then left to saturate for approximately 20 h. Subsequently, they were dried in an oven at 80 °C for 14 h. Following the cleaning process, the samples were fully saturated and aged in formation brine for 7 days, followed by crude oil aging for 21 days to alter their surface wettability to reservoir conditions. All aging processes were carried out at 120 °F and atmospheric pressure. This procedure ensured that the rock samples closely simulated the wettability conditions of the reservoir prior to the contact angle measurements.

### 2.7 Coreflooding Testing

For the displacement tests, a Vinci Technologies CFS 700 coreflooding system was used. This system allows setting a confining pressure and maximum pore pressure of 10,000 psi (with a recommended minimum pore pressure of 500 psi, below the confining pressure) and a maximum furnace temperature of 150 °C. It has two injection pumps that can operate at constant pressure or flow and in a double configuration, providing continuous flow without pulsations, with flow rates ranging from 0.001 to 50 mL/min. The core holder accommodates samples from 1.5″ to 12″ in length. The coreflooding system used is illustrated in Figure 2.

**Figure 2:**
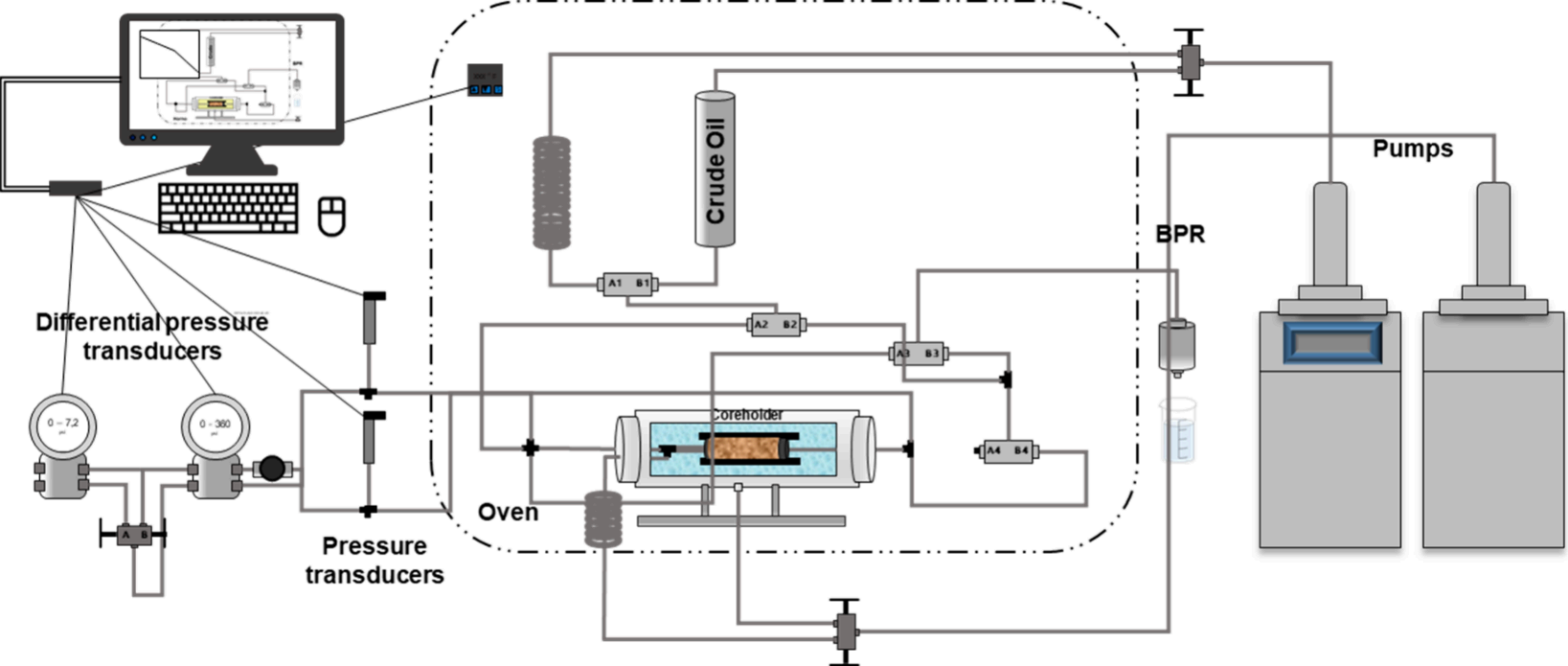
Representative schematic of the coreflooding system used for the test.

#### 2.7.1 Evaluation Protocol for GO in EOR Coreflooding Tests

Coreflooding tests are linear fluid displacement experiments through rock (one-dimensional) conducted at reservoir pressure and temperature conditions. These tests allow for the evaluation of external fluid application effects. To measure crude oil recovery, displacement efficiency (ED) is defined as a measure of how well the displacing fluid mobilizes the oil once in contact in the experiment. Mathematically, it is defined as the ratio of recovered oil to original oil in the rock sample during a waterflooding process. The concept of the recovery factor is avoided as it does not consider heterogeneity, which defines how the reservoir is swept both areally and vertically. In this test, GO was evaluated as a secondary recovery method. The execution protocol was as follows:

a. Rock sample cleaning using solvent extraction (toluene–methanol mixture) and porosity and air permeability measurement using automated equipment.
b. Fluid preparation. Formation brine was filtered through 0.45 μm and deaerated; crude oil was filtered through 0.45 μm.
c. Vacuum saturation of the rock sample with formation brine and pore volume determination, ensuring saturation above 95% compared to the pore volume measured in step b.
d. Sample mounting in the equipment and thermal expansion at test conditions for 12 h.
e. Injection of formation brine to determine absolute water permeability (*K*_w_). A stability criterion of 10 stable pore volumes was used.
f. Injection of formation crude oil to determine effective oil permeability (*K*_eo #1_) and quantification of the expelled brine volume for irreducible water saturation (*S*_wirr #1_) calculation.
g. Sample dismounting and static wettability restoration by placing the sample in a cylinder with crude oil at pressure and temperature conditions for 21 days.
h. Sample remounting in coreflooding equipment at pressure and temperature conditions, displacement of formation crude oil to determine effective oil permeability (*K*_eo #2_), and quantification of the expelled brine volume to recalculate irreducible water saturation (*S*_wirr #2_).
i. Injection of formation brine to determine effective brine permeability (*K*_ew #1_), quantification of the expelled oil volume over time for residual oil saturation (*S*_or #1_) calculation, and pressure differential over time for relative permeability curve calculation using history matching. The base ED was calculated.
j. Injection of formation crude oil to determine effective oil permeability (*K*_eo #3_) and quantification of the expelled brine volume to recalculate irreducible water saturation (*S*_wirr #2_). At this point, *K*_eo #2_ and *K*_eo #3_ were expected to be very similar.
k. GO injection, quantification of the expelled oil volume over time for residual oil saturation (*S*_or #2_) calculation, pressure differential over time for relative permeability curve calculation using history matching, and displacement efficiency determination.

## 3 Results and Discussion

### 3.1 Characterization of the GO

Figure 3 shows the FTIR spectrum of the GO, revealing the presence of oxygen functional groups characteristic of GO. A band at 3285 cm^–1^ is characteristic of the stretching of hydroxyl groups (−OH). At 1729 and 1627 cm^–1^, two bands appear, which are attributed to the stretching vibration of carbonyl (C=O) and (C=C) bonds, respectively. At 1350 cm^–1^, a band corresponding to the stretching of the C–OH bond is found. At 1121 and 1020 cm^–1^, there are two bands associated with the C sp^2^–O and C sp^3^–O stretching vibrations, respectively.

**Figure 3:**
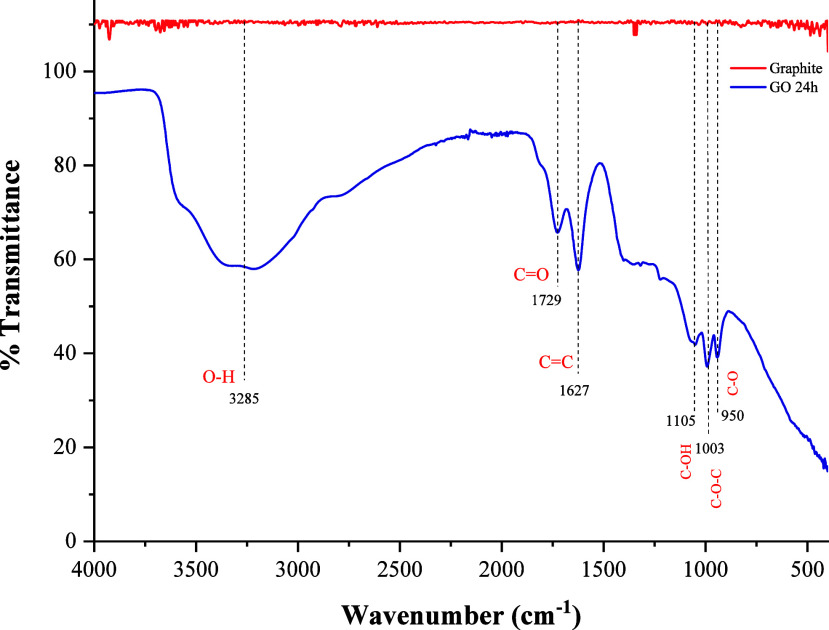
FTIR spectra of GO.

The size distribution analysis (Figure 4) was performed using the optimized nanofluid formulation consisting of 900 ppm GO dispersed in 900 ppm formation brine at pH 8, consistent with the stability results described in Section 2.5. It shows that the GO is in the range from 100 to 400 nm with an average diameter of 220 nm. The zeta potential, shown in Figure 5, has a negative value, suggesting that the dispersion is stable and has an affinity for electropositive substances.

**Figure 4:**
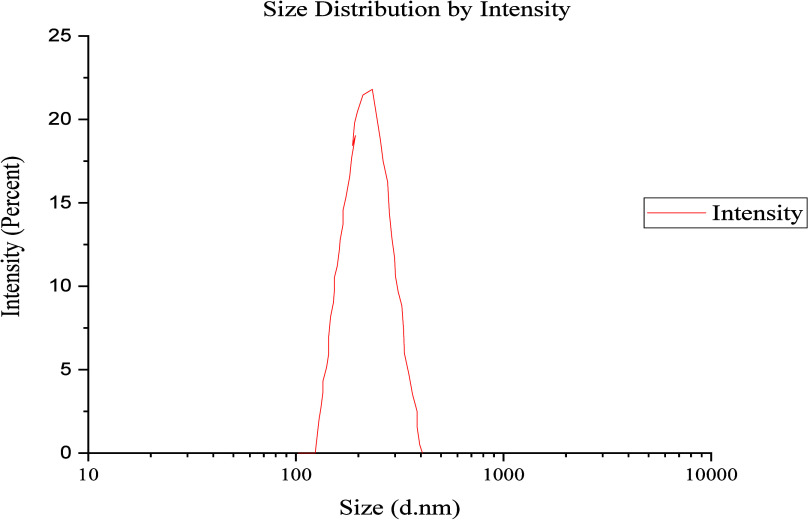
Particle size distribution of GO measured by DLS using the optimized nanofluid (900 ppm GO + 900 ppm brine, pH 8).

**Figure 5:**
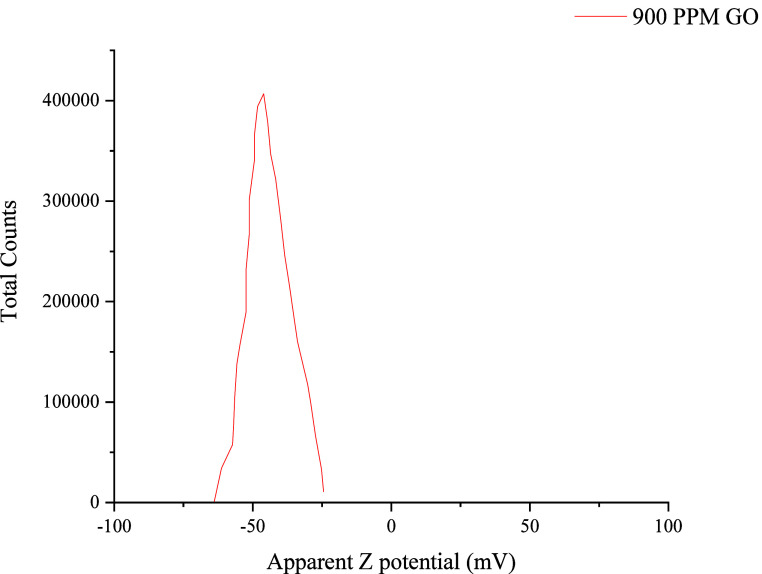
Intensity-weighted particle size distribution of GO measured by DLS using the optimized nanofluid (900 ppm GO + 900 ppm brine, pH 8).

The morphology and some structural characteristics of GO were analyzed by TEM. Figure 6a–c shows representative images of GO. The typical conformation is observed, in which thin sheets with an extension of hundreds of nm^2^ are folded like a silk veil over the copper sample holder (lacey grid). Figure 6 displays thin, wrinkled GO sheets with irregular edges. The sheets are overlapping and folded, which is typical of GO due to its flexible nature and the presence of oxygen-containing functional groups.

**Figure 6:**
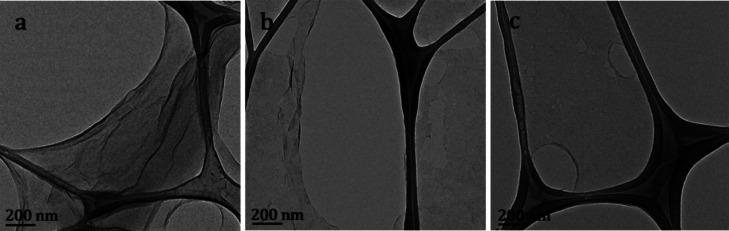
TEM images (a–c) of GO sheets.

Finally, a thermogravimetric analysis for GO was performed. The TGA profiles observed in Figure 7 show a weight loss of 30.9% for GO at 100 °C. This is generally attributed to the desorption of water molecules, though some authors note that decomposition of epoxide groups accompanied by CO_2_ release also occurs.^22^ The most significant mass loss is observed in the range of 180–250 °C, resulting from the decomposition of labile oxygen groups into CO_2_, CO, and H_2_O.^12^ This process, accompanied by rapid gas release, is typically associated with thermal expansion of the material; for this reason, it is recommended to consider the sample quantity in relation to the crucible size. Analyzing the GO profile, it is observed that it is thermally less stable compared to graphite, precisely due to containing more oxygenated functional groups in its structure. After 280 °C, the rate of mass loss is practically constant, and it is considered that after prolonged degradation, the structure of GO resembles that of humic acids.^23^

**Figure 7:**
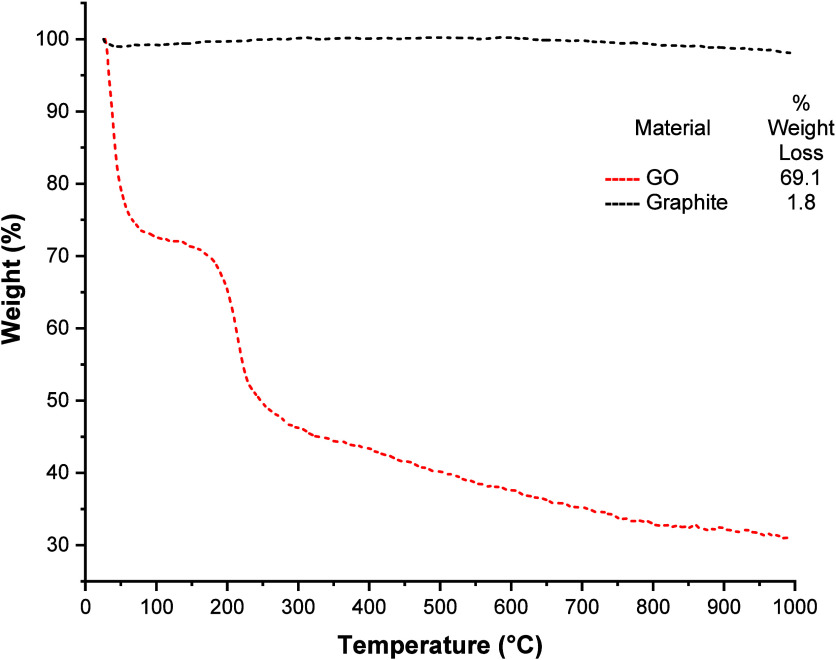
TGA analysis of GO.

### 3.2 Interfacial Tension and Contact Angle Measurements (IFT)

IFT and contact angle measurements were performed at different concentrations of nanofluids and different pH as mentioned above, in 900 ppm formation brine solutions to choose the best formulation at the best concentration. The results show that the IFT values decreased for the solution at pH 8 and the highest concentration of GO (0.09 wt %) as shown in Figure 8. Although the results are not ultralow, there was a 50% reduction in IFT without the addition of surfactants or chemical additives considering that it is a heavy crude oil. This reduction in IFT can be explained by the interfacial adsorption of GO nanosheets, whose amphiphilic structure and oxygen-containing functional groups facilitate their partial alignment at the oil–water interface, lowering interfacial free energy and stabilizing the interfacial film. The lowered surface tension between the fluids can make it easier to push out the trapped oil, improving accessibility for extraction. This enhancement in oil recovery rates is vital for maximizing output in aging reservoirs.^24^

**Figure 8:**
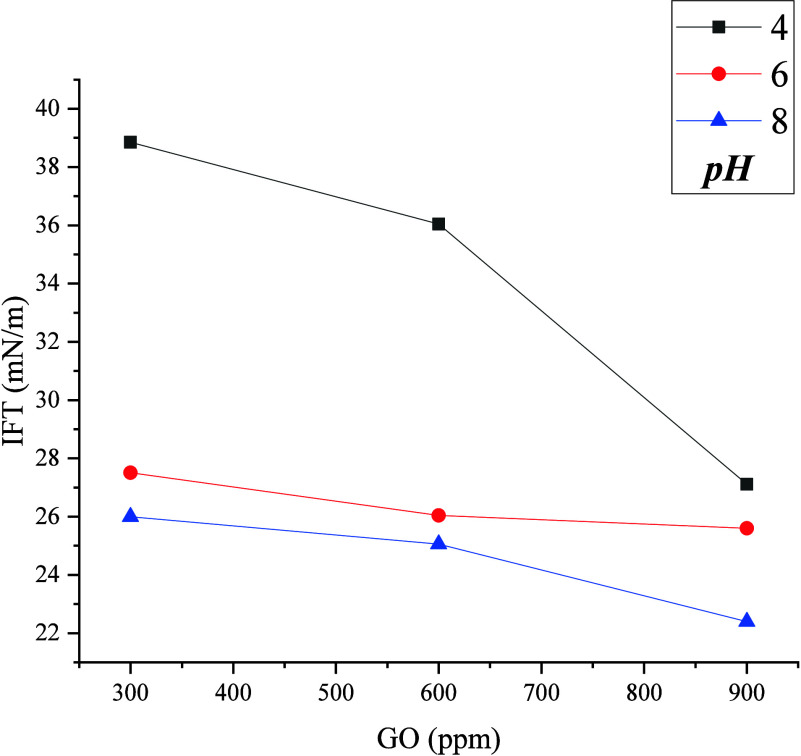
IFT of the GO at different pH.

These results show an IFT reduction from 32.5 to 15.8 mN/m under optimal conditions, which is comparable to Liu et al.,^11^ who observed a significant decrease in interfacial energy using an amphiphilic graphene oxide derivative (GOA) at concentrations as low as 45 mg/L. While these results do not reach the ultralow IFT values (12.2 mN/m) achieved by Cao et al.^13^ with a polyoxyethylated graphene oxide-based nanofluid (P-GO-O), it is noteworthy that this study evaluates unmodified GO, suggesting that further optimization and functionalization could enhance its performance. Additionally, the reduction in IFT observed is also comparable to Li et al.^25^ who developed a fluorescent carbon nanoparticle-based nanofluid that reduced the oil–water interfacial tension to 13.4 mN/m and showed excellent antitemperature, antisalinity, oil displacement, and wettability alteration properties. This comparison highlights the promising potential of graphene oxide in EOR applications.

The more pronounced reduction in IFT at pH 8 is associated with the increased deprotonation of GO’s oxygenated functional groups, which enhances the negative surface charge of the nanosheets. This promotes stronger electrostatic repulsion and better dispersion stability, facilitating the adsorption and partial alignment of GO at the oil–water interface, thereby lowering the interfacial free energy more effectively at high pH. Since the ionic strength was kept constant for all measurements, the sharper IFT reduction is primarily governed by pH-induced modifications of GO surface charge rather than by brine ion dissolution.

Regarding the contact angle, a change from approximately 140° (strongly oil-wet) to 90° (intermediate conditions) was observed, as shown in Figure 9. This alteration can be attributed to the behavior of graphene oxide when dispersed in water. The carboxyl (−COOH) groups, and likely the hydroxyl (−OH) groups, undergo ionization. This process releases protons (H^+^), resulting in a dispersion with a pH value below 7 (acidic pH). The deprotonation of graphene oxide enhances its stability in an aqueous medium due to electrostatic repulsion between the flakes.^20,21^ However, an increase in the salinity of the medium can lead to interaction between the salt cations and the carboxylate groups of the graphene oxide. This interaction neutralizes the charge, allowing interflake interactions, which could result in agglomeration and subsequent destabilization of the dispersion. One method to restabilize graphene oxide in the presence of salts is to increase deprotonation by elevating the pH.

**Figure 9:**
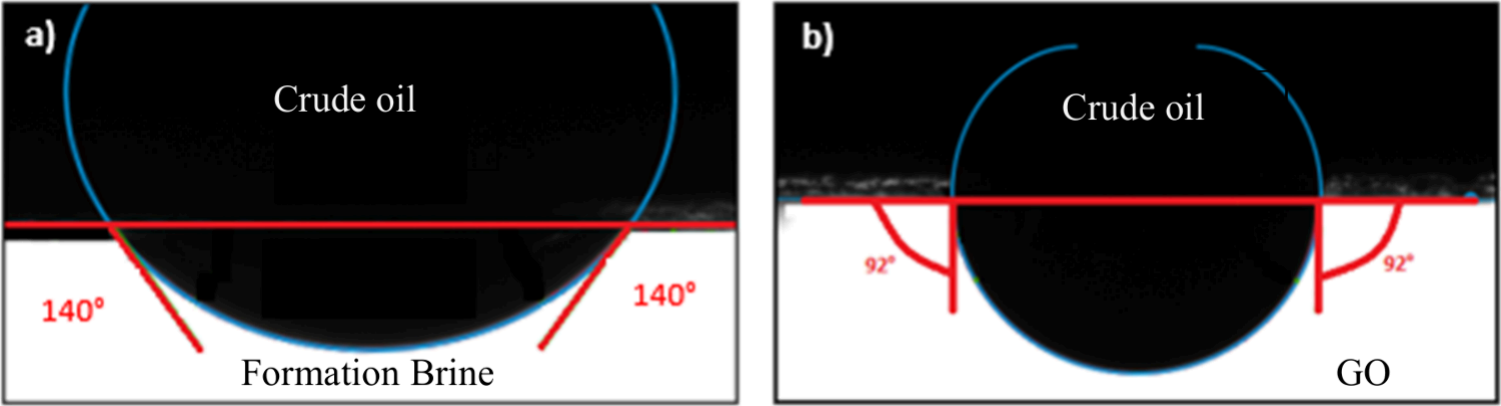
Contact angle measurement: (a) white test and (b) optimal condition.

The observed alteration of the contact angle from 140° (oil-wet) to 90° (intermediate wettability) is significant, indicating a favorable change in wettability even without functionalization. This demonstrates the potential of unmodified GO to alter wettability under specific reservoir conditions. Comparatively, Cao et al.^13^ achieved a complete shift to water-wet conditions using functionalized GO.

### 3.3 Coreflooding Testing

The displacement tests were conducted using a sandstone core sample measuring 3 in. in length and 1.5 in. in diameter. Prior to testing, the core was thoroughly cleaned to remove any residual hydrocarbons or contaminants. Then, the plug was saturated with synthetic brine formation, filtered by a membrane (0.45 μm), and deserted; the saturated plug is reweighed; Table 3 shows the data before and after saturating the plug to ensure that the plug remains completely saturated.

**Table 3:** Saturated Plug Data

setting	value
sample name	B-M-GO-1
length (cm)	7.16
diameter (cm)	3.77
dry weight (g)	164.87
brine density (g/mL)	0.9968
saturated weight (g)	182.50
saturation (%)	100

The coreflooding is assembled, and the working conditions are set as ambient temperature, with a confining pressure of 1000 psi and a BPR of 200 psi; then, absolute permeability is measured resulting in 520.20 mD at a rate of 1 cm^3^/min with an associated pressure differential of 0.3 psi. Subsequently, thermal expansion was initiated at the operating temperature (*T* = 52 °C) for 12 h, replicating the actual reservoir conditions of the fluids used. The confining pressure (1000 psi) and backpressure regulator (BPR) pressure (200 psi) were maintained throughout this period. After this time, the absolute permeability was measured at the operating temperature. Next, crude oil was injected at a rate of 1 cm^3^/min in the production direction until *S*_wirr_ was reached, which in this case was 29.54%. The *K*_eo #1_ was then measured, yielding 358.19 mD with an associated differential pressure of 41.9 psi.

Then, the plug is mounted in a free piston with crude to restore the wettability of the sample to reservoir conditions. After restoring the wettability of the plug to reservoir conditions, the sample is mounted in the coreflooding equipment, establishing the working conditions. Once again, crude oil is injected at a rate of 1 cm^3^/min in the production direction and effective permeability is measured postrestoration (*K*_eo #2_), giving 366.05 mD (Figure 10).

**Figure 10:**
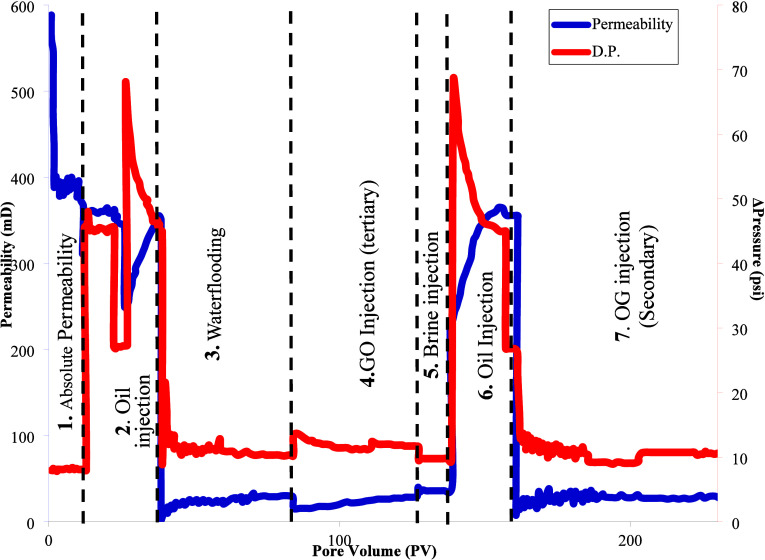
Permeability behavior.

Then, the brine is displaced in the production direction (waterflooding) until the sample is brought to residual oil saturation (*S*_or_). Throughout the process, the produced oil volume (Np), differential pressure (Δ*P*), and time (*t*) were continuously monitored. These data were used to construct relative permeability curves through history matching. Additionally, the displacement efficiency was determined, and the *K*_ew #1_ was measured. In this case, *K*_ew #1_ was found to be 30.32 mD, with an associated differential pressure of 2.75 psi, measured at a flow rate of 1 cm^3^/min. This led to the determination of a residual oil saturation of 30.48%.

During the injection of synthetic brine representative of the formation brine (waterflooding), the oil produced was monitored (Figure 11), which was determined by material balance. As shown in the revised Figure 10, the GO injection stage (stage 4) exhibits a slight initial increase in differential pressure, related to the early adsorption of GO on pore surfaces, followed by a progressive decline toward a stable value. This trend does not indicate plugging or face-plugging, and the effective permeability to brine remained unchanged after GO injection. It is important to note that the injection rate was kept constant during the entire GO injection stage, confirming that the observed pressure response corresponds exclusively to fluid–rock interaction. In the injection of synthetic formation brine, a recovery of 7.09 mL of crude oil was achieved, which is equivalent to a microscopic displacement efficiency of 56.72% (Figure 12); it should be noted that the initial oil saturation is 70.46%. Subsequently, the injection fluid was switched to crude oil, which was injected until a stable associated differential pressure was achieved. This process resulted in only a 2.2% change in the effective permeability to crude oil, increasing from 358.19 mD (*K*_eo #2_) to 366.05 mD (*K*_eo #3_). These values are very similar, as expected, given that the injection of formation brine does not induce any alteration in the crude oil–rock–brine system or cause a change in wettability, reaching an initial oil saturation for this case of 72.8%, and evaluate the injection of the GO nanoparticles in secondary mode. The viscosity analysis indicated that the graphene oxide nanodispersion exhibited no significant deviation from the baseline viscosity of the brine. The methodology applied included using a single rock sample to avoid mineralogical changes, which can be sensitive in evaluating the technology. Even twin samples can present variations that would condition the saturations within the plug and could lead to masking some results. Therefore, a single sample was used to first evaluate conventional waterflooding, then resaturate with crude oil, and finally evaluate GO as a secondary recovery method. This approach helps isolate the effects of the GO treatment by using the same rock sample as its own control. It eliminates variability from sample to sample that could confound the results. By conducting waterflooding first, then resaturating and testing GO flooding on the same core, direct comparisons can be made while keeping the rock properties constant. This allows for a more accurate assessment of GO’s effectiveness as an enhanced oil recovery agent.

**Figure 11:**
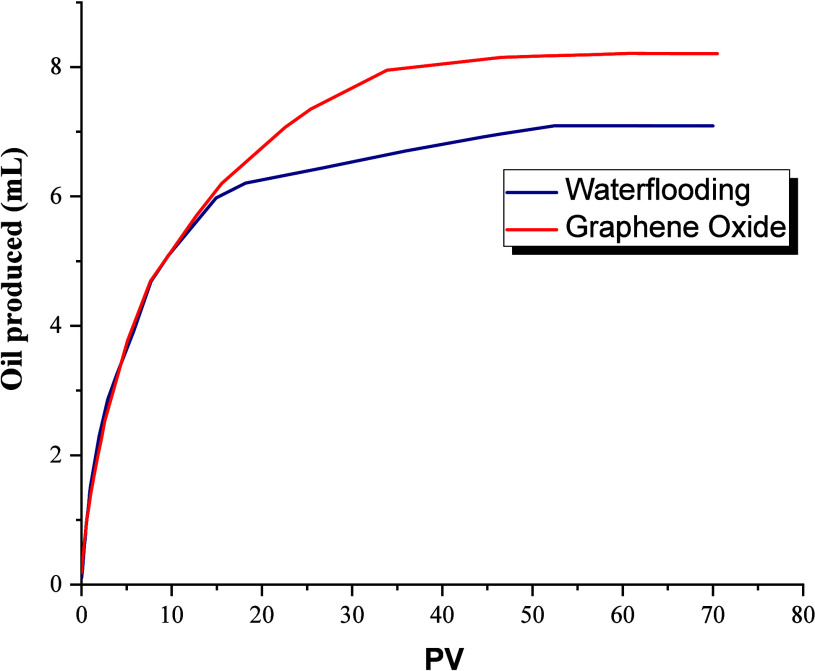
Oil produced.

**Figure 12:**
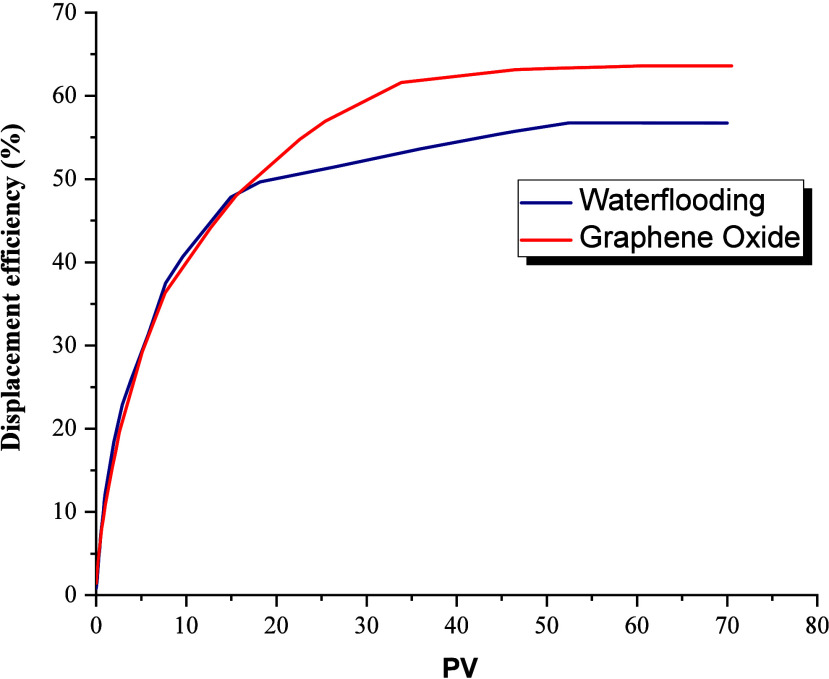
Displacement efficiency.

The evaluation of the GO formulation in secondary mode allowed obtaining a higher crude oil production, reaching 8.21 mL, as can be seen in Figure 11, being this in terms of displacement efficiency 63.60%, that is, approximately 7% more compared to waterflooding (Figure 12), finally achieving a residual oil saturation of 26.48%.

The 7% incremental oil recovery achieved in this study, while modest compared to some reports, is significant considering the challenging conditions of Colombian heavy-oil reservoirs. It is comparable to the 7.8% improvement reported by Aliabadian et al.^12^ using GO functionalized with OH groups in combination with partially hydrolyzed polyacrylamide solutions. The lower incremental recovery obtained in this study compared with the results reported by Liu et al., Cao et al., and Aliabadian et al. can be attributed to fundamental differences in crude oil properties, reservoir temperature, nanoparticle formulation, and rock mineralogy. Our experiments were conducted using a heavy crude oil (11–13° API) at a reservoir-representative temperature of 52 °C and with unmodified GO. Under these conditions, the extent of interfacial tension reduction and wettability alteration is inherently lower than in studies using functionalized GO or operating at higher temperatures. Moreover, the cited studies typically involve lighter oils, temperatures between 70 and 120 °C, and amphiphilic or polymer-modified GO formulations, all of which enhance stability and intensify EOR mechanisms. Additionally, our sandstone core is predominantly quartz-rich, whereas carbonate or mixed-mineral systems reported in the literature show stronger GO–rock interactions. These combined factors explain why the recovery factor observed in our system is lower while remaining fully consistent with the expected behavior for heavy-oil, moderate-temperature, unmodified-GO applications.

The incremental recovery can be attributed to a possible alteration in rock wettability and improved fluid behavior in the porous medium, which is evidenced in the relative permeability curves. Figure 13 shows how the intersection of the graphs shifts to the right, which, according to Craig’s rules, is indicative of increased water wetness. Additionally, the figure presents a series of points that allow for analysis using indices more novel than Craig’s rules.

**Figure 13:**
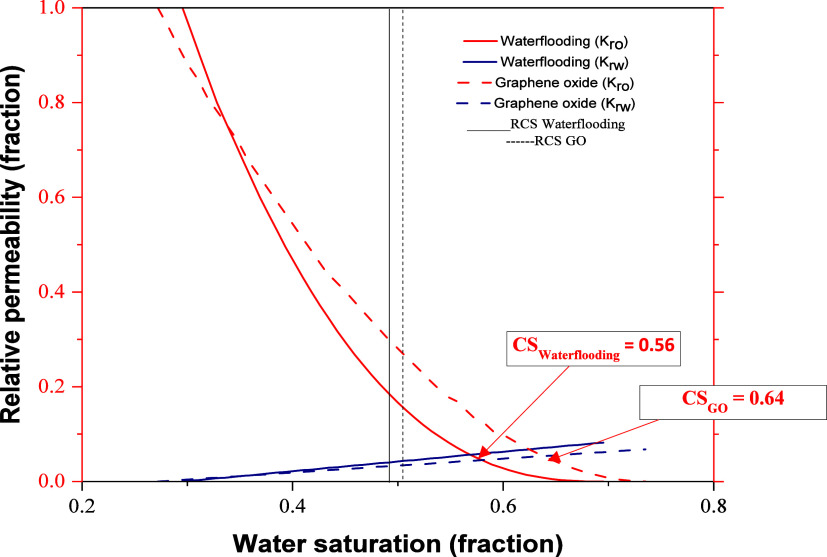
Relative permeability curves.

Craig’s method was for a long time a set of rule-of-thumb guidelines that allowed for the analysis of end points of relative permeability curves and provided an indication of the wettability state of the crude oil–brine–rock system. These rules were based on the values that could be obtained from interstitial water saturation, oil saturation at the crossover point of relative permeability curves, and relative permeability to water at residual oil saturation. In this research, the Lak and modified Lak indices, proposed by Mirzaei-Paiman (as a modification to Craig’s rules), will be used to quantitatively establish the degree of wettability according to a pair of relative permeability curves. For the first index, the RCS (reference crossover saturation) was defined and compared with the curve crossover point (CS). For the second index, the author proposes comparing the areas under each of the curves. Both indices range from −1 to 1, where the lower value represents a strongly oil-wet system and the higher value represents a strongly water-wet system, while values close to zero indicate neutral wettability.^26,27^ Table 4 summarizes the equations associated with each index.

**Table 4:** Indices for Quantifying Wettability Characteristics

Lak index (*I*_L_)		modified Lak index (*I*_ML_)
$I_{L}=\alpha (\frac{0.3-k_{\mathrm{rw}}@S_{\mathrm{or}}}{0.3})+\beta (\frac{0.3-k_{\mathrm{rw}}@S_{\mathrm{or}}}{0.3})+\frac{\mathrm{CS}-\mathrm{RCS}}{1-S_{\mathrm{or}}-S_{\mathrm{wir}}}$		$I_{\mathrm{ML}}=\frac{A_{o}-A_{w}}{A_{o}+A_{w}}$
if	*k*_rw_@*S*_or_ < 0.3, then α = 0.5 and β = 0	*A*_o_, the area under the oil curve
	0.3 ≤ *k*_rw_@*S*_or_ ≤ 0.5, then α = β = 0	*A*_w_, the area under the water curve
	*k*_rw_@*S*_or_ > 0.5, then α = 0 and β = 0.5	
	CS = water saturation at the crossover point	
	$\mathrm{RCS}=\frac{1}{2}+\frac{S_{\mathrm{wc}}-S_{\mathrm{or}}}{2}$	

Table 5 presents the end point values and curve intersection points for both water injection and GO formulation injection scenarios, along with the corresponding Lak and modified Lak indices. While the RCS values remained relatively constant across both injection scenarios (0.495 for water and 0.503 for GO), a notable difference was observed in the CS. The CS shifted rightward during GO injection, indicating an increase in the water wetness of the system. This trend is consistently reflected in both indices studied, with GO injection resulting in values closer to 1, which is indicative of strong water wettability. This observation suggests that the GO formulation enhances the water-wet characteristics of the rock–fluid system more effectively than conventional water injection.

**Table 5:** Experimental Results of Relative Permeabilities and Wettability Alteration

coreflooding	*S*_wirr_	***K*_ro_** @ ***S*_wirr_**	*S*_or_	***K*_rw_** @ ***S*_or_**	CS	RCS	Lak index	modified Lak index
waterflooding	29.5%	1.000	30.5%	0.076	0.56	0.495	0.535	0.748
GO	27.2%	1.000	26.5%	0.062	0.64	0.503	0.691	0.821

### 3.4 Adsorption of GO on the Rock Surface

After the coreflooding test, scanning electron microscopy (SEM) analysis was performed to observe the adsorption of GO nanoparticles on the rock surface under the test conditions. This analysis is crucial for understanding the interaction between the nanoparticles and the porous medium, which directly impacts the EOR process.

Figure 14 presents SEM images of the rock surface before and after GO nanofluid injection. Figure 14a shows the untreated sandstone, while Figure 14b displays the rock surface after treatment. In Figure 14a, we observe a typical sandstone structure with interconnected pores. The initial pore sizes range from approximately 5 to 30 μm, with an average pore throat size of about 12 μm. These large pores play a crucial role in fluid flow through the rock matrix.

**Figure 14:**
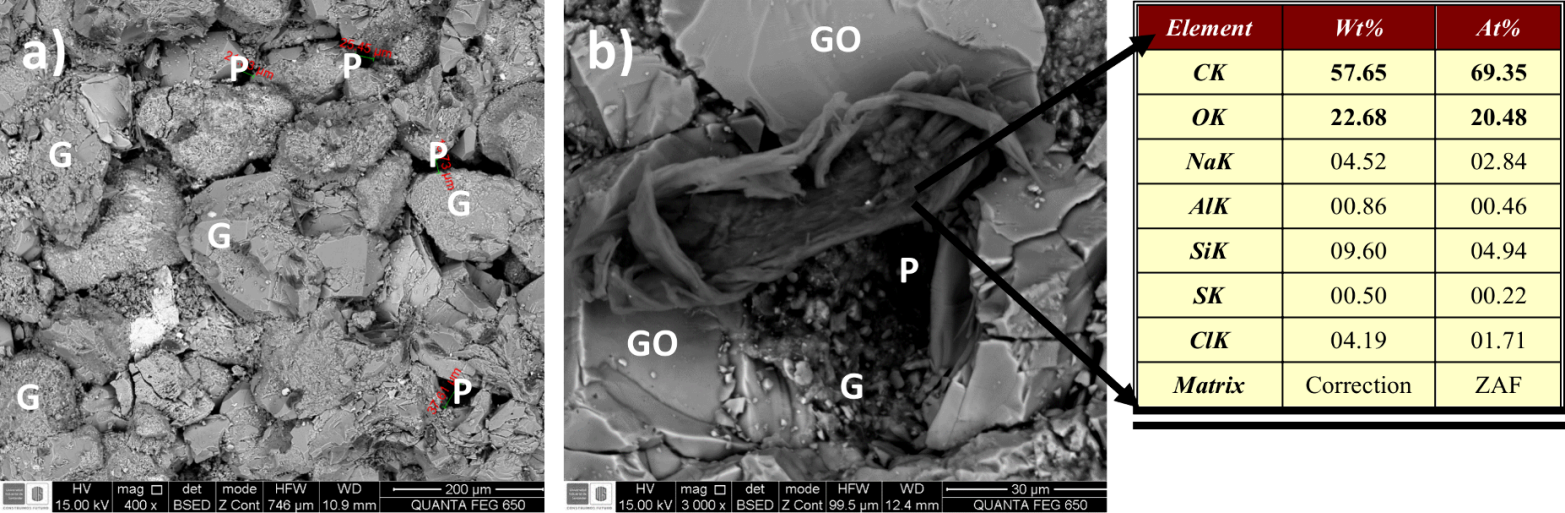
SEM images of sandstone (a) before and (b) after optimal GO nanofluid treatment. G: rock grains, P: pores, AND GO: adsorbed GO nanoparticles.

Figure 14 reveals significant changes in the rock surface morphology after GO nanofluid injection. GO nanoparticles, with an average size of 100–200 nm, are visible as thin, sheet-like structures adsorbed onto the rock grains (labeled as GO). These nanoparticles form a thin film on the grain surfaces (G), altering the surface properties of the rock. Importantly, the large pores (P), some measuring up to 25.45 μm, remain open after GO nanofluid injection. The average pore throat size decreased slightly to 10 μm post-treatment, still well above the average GO nanoparticle size of 150 nm.

This observation is critical as it indicates that the nanoparticles do not significantly reduce permeability during injection, which aligns with the previously presented relative permeability results showing no formation damage. Postflooding absolute permeability measurements confirmed only a 5% reduction from the initial value, further supporting that GO adsorption did not cause significant pore blocking or formation damage. This is consistent with the findings of Radnia et al.,^20,21^ who also observed GO adsorption on sandstone rocks without significant formation damage. This behavior is corroborated in Figure 10, which shows negligible variation in effective brine permeability before and after GO injection (stage 4).

The uniform coating of GO nanoparticles on the rock surface likely alters the wettability of the rock from oil-wet to intermediate conditions. This change is reflected in the displacement efficiency shown in Figure 12. The thin GO film visible on grain surfaces is crucial for wettability alteration, as it modifies the rock–fluid interactions without obstructing fluid pathways. This selective adsorption mechanism is key to the EOR potential of GO nanofluids, as it enhances oil mobilization while maintaining formation integrity.

The elemental analysis data provided in the table offer additional insights into the adsorption process: The carbon (C) content increased to 57.85% after treatment, indicating the presence of adsorbed carbon-rich GO nanoparticles. The oxygen (O) content of 22.68% might be due to the interaction between GO and the rock surface, as well as the oxygen-containing functional groups in GO. The presence of silicon (Si) and aluminum (Al) confirms the sandstone composition. Trace elements like sodium (Na) and chlorine (Cl) are also detected, which may be present due to the brine content in which the nanofluid was prepared.

The relative permeability measurements (Figure 13) corroborate the SEM observations, confirming that GO nanoparticle adsorption does not significantly obstruct fluid flow paths. In fact, the data suggest that GO nanofluid injections may enhance fluid mobility and displacement efficiency. This improvement can be attributed to the wettability alteration induced by the adsorbed GO nanoparticles, which facilitates the release of trapped oil from the pore spaces without compromising the rock’s permeability.^28–30^

### 3.5 Numerical Simulation

#### 3.5.1 Simulation Process

To explore the potential behavior of GO nanofluid injection at the reservoir scale, a conceptual approach is proposed through the implementation of a numerical model in the STARS simulator, part of the software package developed by Computer Modeling Group (CMG). This simulation seeks to project the impact that this technology could have in representative subsurface conditions, based on the experimental results previously obtained in the laboratory.

#### 3.5.2 Laboratory-Scale Simulation

To analyze the experimental results obtained and model the behavior observed in the laboratory, a numerical simulation of the experiment is performed using the CMG-STARS simulator, where, in order to adequately represent the rock sample used, a mesh is defined (Figure 15) whose geometric characteristics and petrophysical properties are dictated by those of the core used; Likewise, the fluids involved are loaded through the component section of the simulator, where the required properties are established to represent both the fluids present and those injected into the rock, i.e., oil, water, and the GO nanofluid.

**Figure 15:**
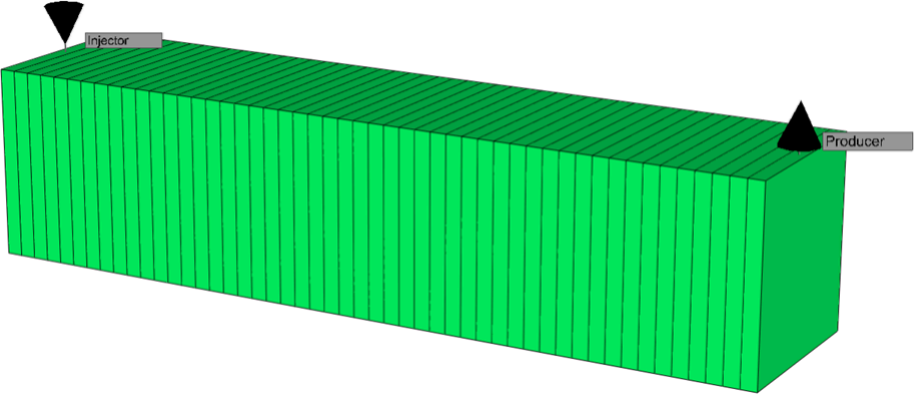
3D view of the laboratory-scale simulation crew.

Similarly, a set of initial relative permeabilities are also defined for the smart water system; such a set of permeabilities will be adjusted to correctly represent the displacement observed experimentally; achieving this adjustment means obtaining the simulation parameters that represent the behavior associated with the injection of the GO nanofluid.

The refinement of the relative permeability curves is performed by means of a historical adjustment in the CMOST AI module of CMG, where, starting from the creation of a historical file that stores the crude recovery results obtained in the coreflooding test and the selection of the simulation model parameters that will act as iteration variables, the optimization process that allows adjusting the relative permeability curves is carried out. This procedure contemplates a total of 100 iterations or experiments, at the end of which the case that best represents the trend described by the historical archive is selected.

Figure 16 shows the comparison of cumulative oil production over time, represented by three distinct curves: Initials_Kr (dashed green line), which corresponds to the relative permeability curves used initially; Historical_file (red circles), which represents the historical cumulative production data; and Adjusted_Kr (solid green line), which shows the adjusted relative permeability curves, i.e., the result of the historical adjustment process performed to improve agreement with the observed experimental results.

**Figure 16:**
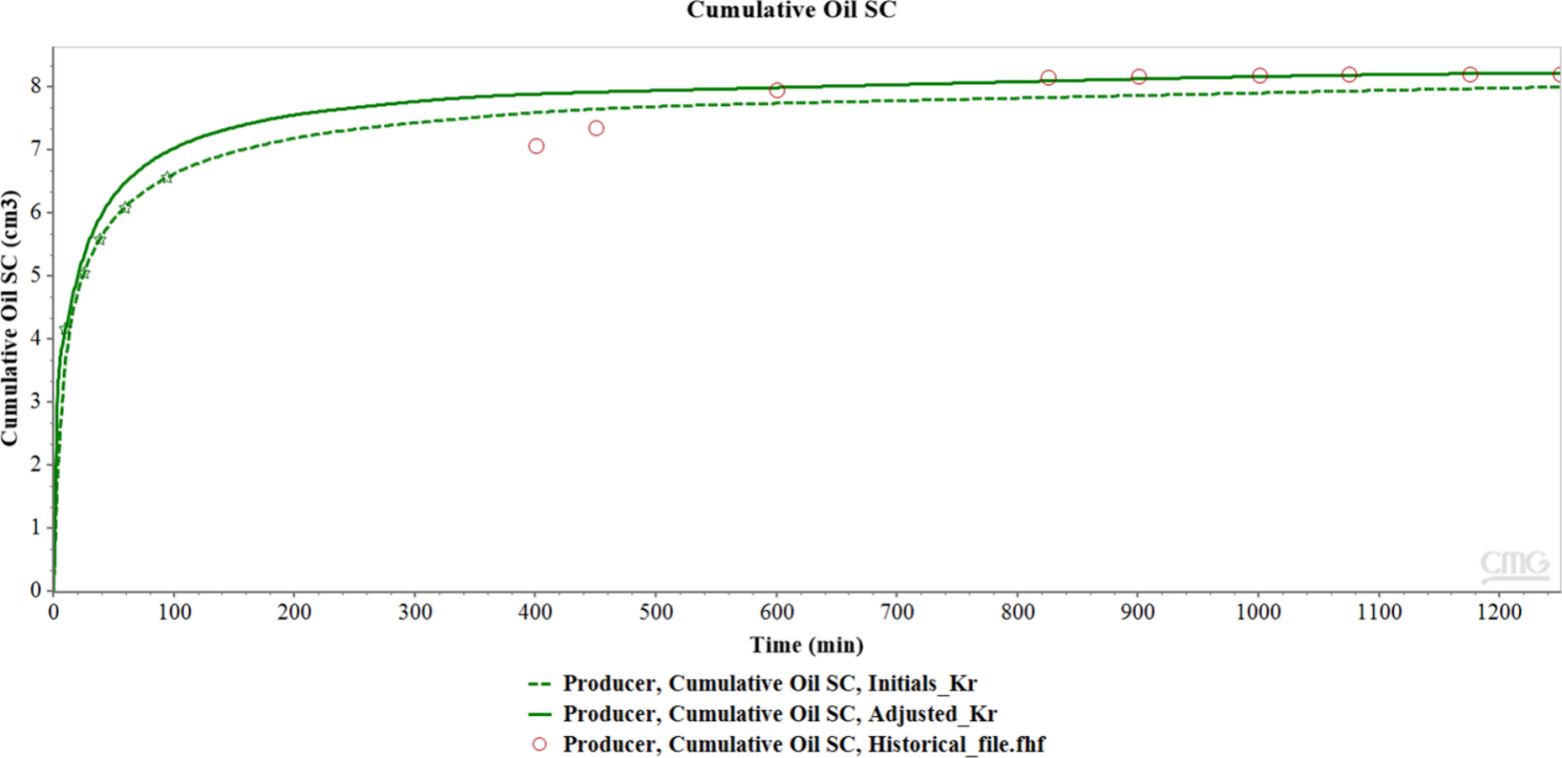
Comparison of the cumulative oil curves of the initial and adjusted relative permeabilities with the historical file.

As can be seen, the adjusted relative permeability curves (readjusted) provide a better match with historical data, especially at later times, indicating an improvement in model accuracy after adjustments. Finally, the estimated relative permeability curves for each system are shown in Figure 17.

**Figure 17:**
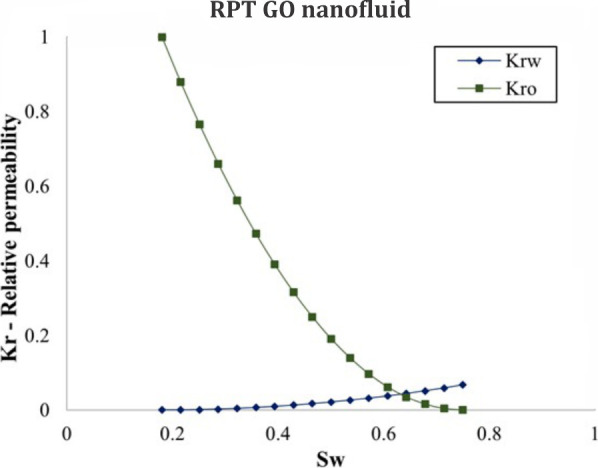
Relative permeabilities are estimated in the simulator for the injection of the GO nanofluid.

#### 3.5.3 Reservoir-Scale Simulation

In other words, the objective is to perform a simulation using the same fluids (components) and permeability curves used in the laboratory scale, but in this case aimed at predicting the possible results that could be obtained from the application of the GO nanofluid injection process at a field level. It is important to highlight that the conceptual model is homogeneous in the areal plane (*X*–*Y*), but heterogeneous in the vertical direction (*K*), where permeability and porosity vary to represent the reservoir layering.

In this order of ideas and with the purpose of achieving a representative recreation of the petrophysical characteristics present in a reservoir, the petrophysical information available from an oil well will be used. Table 6 is presented below with the cell numbers and the area used for the creation of the grid.

**Table 6:** Description of the Mesh Characteristics of the Evaluated Case

area [acres]	40
# of cells *I*, *J*, and *K*	50, 50, and 30
total evaluated thickness [ft]	162.5

The limiting and average values used for permeability and porosity in the model can be seen in Table 7.

**Table 7:** Permeability Limit Values

	permeability [mD]	porosity [%]
minimum value	2.24	9.72
maximum value	557.65	21.78
average	134.05	17.19

The collection and union of this information result in the creation of the following simulation mesh (Figure 18).

**Figure 18:**
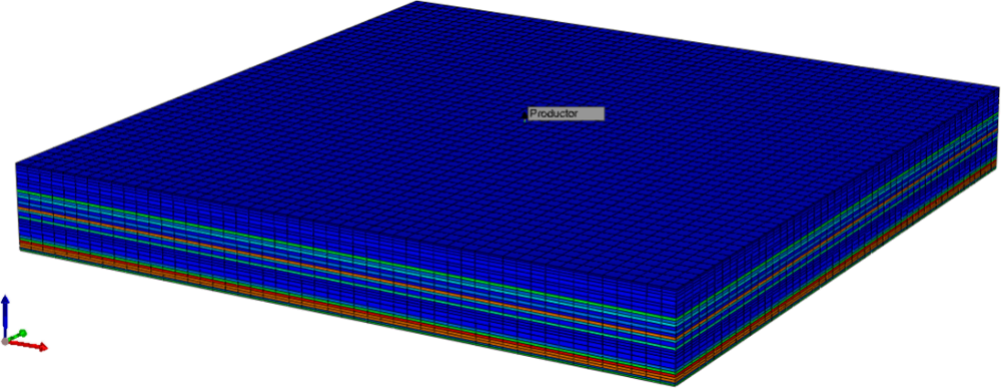
3D view of the conceptual model grid at the field scale.

To conclude this stage, it is necessary to highlight that, in order to evaluate the GO nanofluid injection process as realistically as possible at reservoir conditions, an initial depletion period is simulated in order to simulate a primary production stage in the reservoir, followed by an injection period in the field reflecting the beginning of the water injection processes commonly carried out as a secondary recovery method; in this particular process, a normal 5-point injection pattern was used, with a simulation period that was carried out until the end of the year 2023.

The development of the simulation under this order of events provides a solid basis for the evaluation of the performance of the GO nanofluid injection process. In this context, and with the objective of comparing the impact of GO nanofluid injection versus the conventional water injection process, two simulations recreating both injection methods were carried out, starting on January 2024. Figure 19, presented below, clearly shows the workflow of each simulation, together with the evaluation period used for the comparison of both.

**Figure 19:**
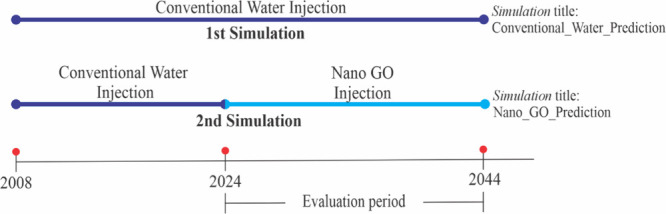
Workflow-handled simulations.

From the results obtained in the two simulations, it is observed that GO nanofluid injections generate considerable improvement in crude oil recovery compared to conventional water injections. This increase in oil production is noticeable immediately after the injection begins, with an increase of 0.875 barrels that continues to increase considerably until the end of the evaluation period. In total, the improvement in production continues throughout the 20-year evaluation period, reaching a total net increase of 402,431 barrels (BLS). Figure 20 clearly shows this trend, highlighting how GO nanofluid injections achieve higher oil recovery than the conventional method.

**Figure 20:**
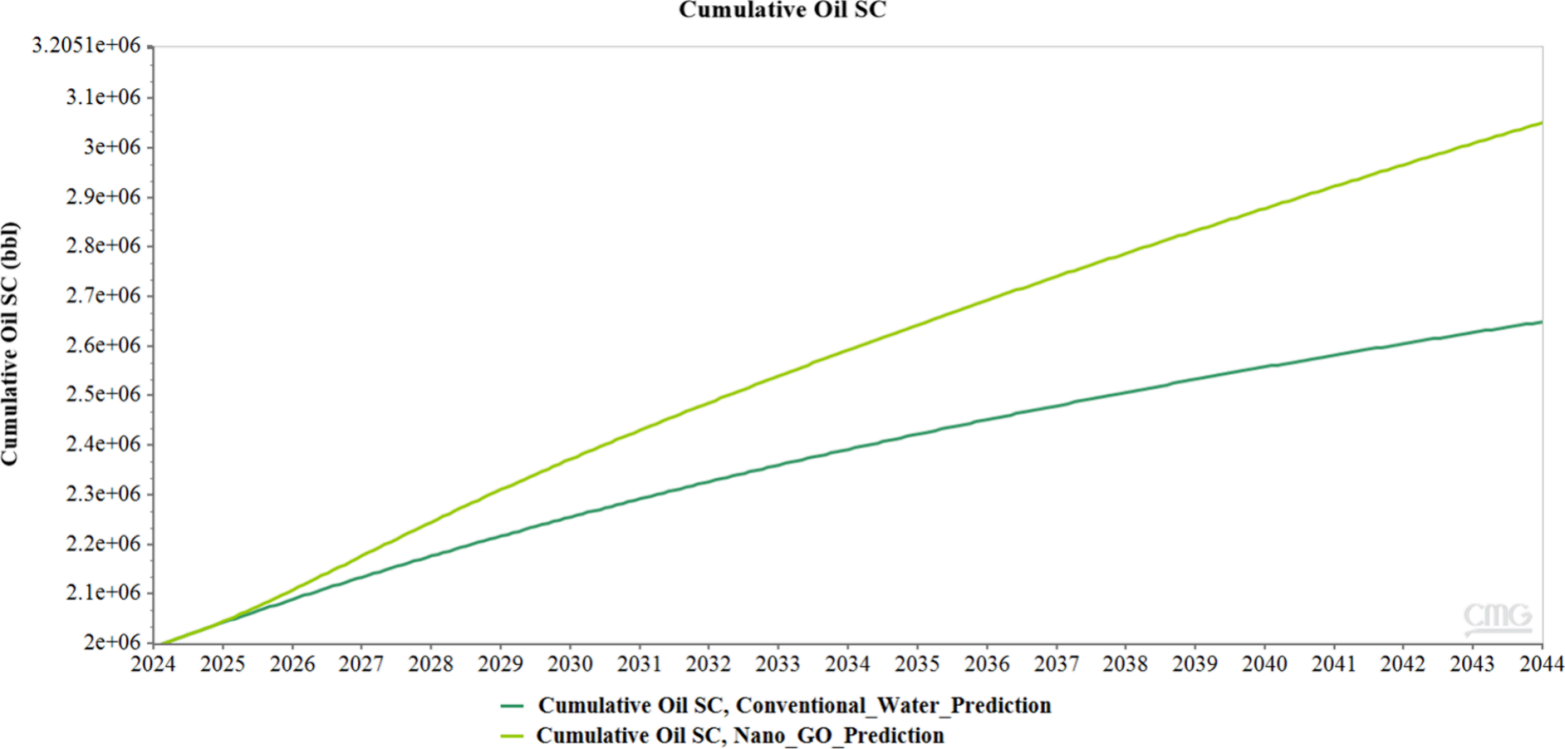
Oil production graph for conventional water and GO nanofluid simulations.

Furthermore, in terms of water production, it can be identified that the injection of the GO nanofluid generates a significant increase in water production compared to conventional water injection; in total, the said increase in water production corresponds to 2,552,560 barrels. However, despite such an increase, Figure 21 corresponding to the water cut shows that the curve associated with the injection of the GO nanofluid remains consistently lower than that reported by conventional water injection throughout the entire analyzed period, which shows a more favorable performance in terms of water production control. This behavior suggests that, although more water is produced in absolute terms, the injection of the GO nanofluid allows a more effective recovery of oil, distributing the injection more efficiently and allowing production with a lower water fraction for a longer time, which is favorable from an economic and operational point of view.

**Figure 21:**
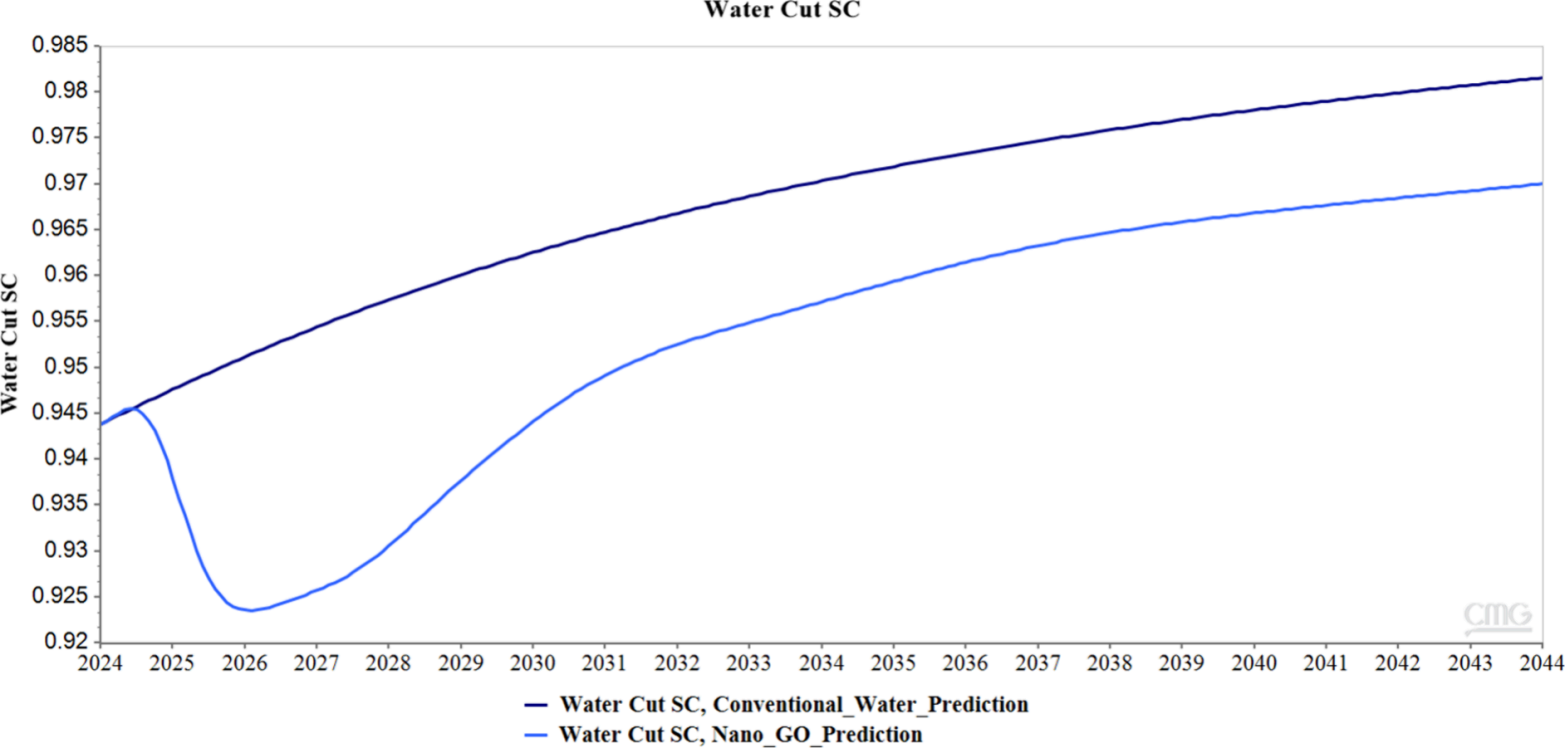
Water cut graph for conventional and GO nanofluid water simulations.

## 4 Limitations of the Study and Future Research Direction

### 4.1 Study Limitations

Controlled experimental conditions: The results of this study rely on a meticulously planned experimental protocol carried out under controlled laboratory conditions, which may not fully replicate the heterogeneous and dynamic conditions of actual oilfields. Variables such as temperature, pressure, and crude oil composition can vary significantly in natural environments, potentially affecting the performance of graphene oxide (GO) nanoparticles in enhanced oil recovery (EOR).

Study scale experiments were conducted on the laboratory scale, which may not fully capture the complexity of large-scale reservoir systems. Extrapolating these results to field-scale operations may require additional studies to confirm the viability and effectiveness of using GO nanoparticles.

### 4.2 Future Research Direction

Field-scale studies: It is essential to conduct pilot tests and field-scale studies to validate laboratory findings and adjust GO nanofluid formulations according to specific reservoir conditions. This includes evaluating the impact of geological variability and reservoir heterogeneity on EOR performance.

Parameter optimization: future research should focus on dynamic optimization of parameters such as nanofluid concentration, pH levels, and salinity in real time during injection. This could include developing predictive models to adjust operational conditions optimally in response to observed changes in the field.

Development of improved materials: Future research could explore the modification and functionalization of GO nanoparticles to further enhance their effectiveness and stability under extreme reservoir conditions. This may include developing GO hybrids with other nanostructured materials to enhance oil recovery properties.

## 5 Conclusions

The conclusions of the study reveal the impact of injecting a GO-based nanofluid at pH 8 on interfacial tension (IFT) reduction from 32.5 to 15.8 mN/m under optimal conditions (900 ppm formation brine, pH 8, and 0.09 wt % GO concentration). This enhancement is attributed to the distinct influence of pH on the charge characteristics of ions present at the GO surface. These results contribute valuable insights into the nuanced understanding of pH-dependent interfacial properties, emphasizing the importance of pH optimization for applications involving GO in scientific and technological domains.

The nanofluid proved to be highly effective. Coreflooding experiments revealed that GO nanofluid injection achieved 63.60% oil recovery, compared to 56.72% with conventional waterflooding, representing a 7% incremental oil recovery. This underscores the promising potential of GO nanofluids in enhancing oil recovery processes.

SEM analysis confirmed the adsorption of GO nanoparticles on the rock surface without significant pore blocking, supporting the observed wettability alteration and improved oil recovery.

The presence of hydroxyl (OH) groups in GO and their interaction with silanol groups underscore the material’s potential as a powerful tool for modifying surface properties, especially in the context of enhanced oil recovery (EOR). The silanol groups present in quartz, along with the siloxane-based facets, play a crucial role in the material’s interaction with crude oil. The oxygen-containing groups on the GO sheets can undergo ionization at higher pH levels, affecting the hydroxyl (OH) groups in sandstone. This dynamic presents promising avenues for EOR strategies. Additionally, the potential for n−π interactions between the oxygen (O) groups of sandstone and the π electrons of GO provides valuable insights into the intricate interfacial mechanisms relevant to improved crude oil extraction. These insights pave the way for exploring innovative approaches and techniques in wettability alteration and surface engineering, specifically designed to enhance oil recovery processes.

GO nanofluid injection produces a significant improvement in oil recovery compared to conventional water injection, resulting in a net additional recovery of 402,431 barrels at the end of the simulation period. These results demonstrate the effectiveness of nano-GO injection in improving displacement efficiency and mobilizing additional oil that would otherwise remain unrecovered.

While GO nanofluid injection produces a higher total volume of produced water as evidenced, the water cut curve remains consistently below that reported for conventional water injection throughout the evaluation period, indicating that the nano-GO injection strategy maintains a more favorable oil-to-water production ratio, which offers advantages from both an economic and operational perspective, as a reduced and delayed water cut suggests that the nanofluid promotes a more uniform displacement front, delaying water breakthrough and allowing for a more sustained and efficient oil production phase.

## Data Availability

All data supporting the findings of this study are available within the article and its Supporting Information.
